# Dysgenesis of the posterior segment of the corpus callosum: don't miss SPG45!

**DOI:** 10.1055/s-0043-1763299

**Published:** 2023-04-14

**Authors:** Daniel de Faria Guimarães, Ana Luiza Viegas de Almeida, Felipe Alba Scortegagna, Renato Hoffmann Nunes, Simone Consuelo Amorim, Felipe Torres Pacheco, Fernando Kok, Antonio José da Rocha

**Affiliations:** 1 Santa Casa de Misericórdia de São Paulo, Faculdade de Ciências Médicas, Departamento de Radiologia, São Paulo SP, Brazil.; 2 Grupo DASA São Paulo, Departamento de Radiologia, São Paulo SP, Brazil.; 3 Clínica Vita, Departamento de Neurologia Pediátrica, São Paulo SP, Brazil.; 4 Universidade de São Paulo, Faculdade de Medicina, Departamento de Neurologia, São Paulo SP, Brazil.; 5 Mendelics Análise Genômica, São Paulo SP, Brazil.

In hereditary spastic paraplegias (HSPs), magnetic resonance imaging (MRI) scans of the brain typically show the involvement of the anterior part of the corpus callosum with abnormalities in the white matter fibers of the forceps minor(“ears of the lynx sign”). However, these imaging findings are particularly associated with spastic paraplegia type 11 (SPG11) or spastic paraplegia type 15 (SPG15). 1

**Figure 1:**
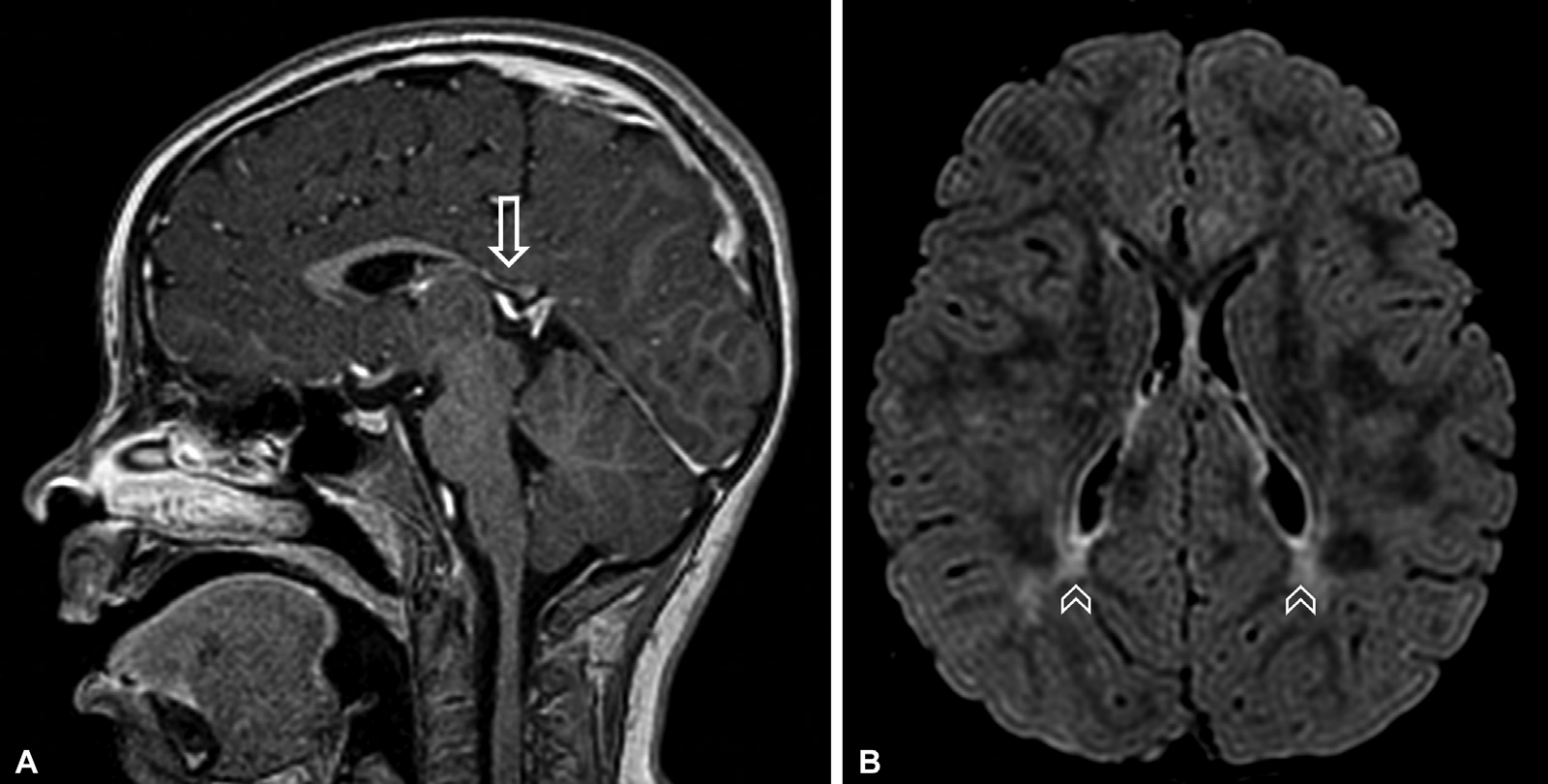
Brain MRI. ( **A** ) Sagittal T1 postcontrast shows hypoplastic corpus callosum with marked reduction of the splenium and posterior segment of its body (arrows), characteristic, even though not specific, of this disease. ( **B** ) Axial fluid-attenuated inversion recovery (FLAIR) demonstrates symmetric and bilateral peritrigonal white matter changes (arrowheads). The ears of the lynx sign is not usually observed in these cases.

A child with spastic gait achieved independent walking at the age of 2 years, and 3 years later was referred to our service for investigation. A brain MRI demonstrated corpus callosum dysgenesis and peritrigonal white matter abnormality ( Figure 1 ). Whole exome sequencing revealed compound heterozygosity for two novel likely pathogenic variants in *NT5C2* (p.[Leu468Pro] and p.[Gln542Argfs*71]), consistent with SPG45, of the SPG subtypes with thin corpus callosum. 2
